# Amide proton transfer (APT) and magnetization transfer (MT) in predicting short-term therapeutic outcome in nasopharyngeal carcinoma after chemoradiotherapy: a feasibility study of three-dimensional chemical exchange saturation transfer (CEST) MRI

**DOI:** 10.1186/s40644-023-00602-6

**Published:** 2023-09-01

**Authors:** Wenguang Liu, Xiao Wang, Simin Xie, Weiyin Vivian Liu, Ismail Bilal Masokano, Yu Bai, Juan Chen, Linhui Zhong, Yijing Luo, Gaofeng Zhou, Wenzheng Li, Yigang Pei

**Affiliations:** 1 Department of Radiology, National Clinical Research Center for Geriatric Disorders, Xiangya Hospital, Central South University, No.87 Xiangya Rd., Kai Fu District, Changsha, 410008 Hunan China; 2 MR Research, GE Healthcare, Beijing, 100176 China; 3 Department of Radiology, The Third Xiangya Hospital, Central South University, Changsha, 410013 Hunan China

**Keywords:** Chemical exchange saturation transfer, Magnetization transfer, Amide proton transfer imaging, Nasopharyngeal carcinoma, Chemoradiotherapy

## Abstract

**Background:**

The three-dimensional chemical exchange saturation transfer (3D CEST) technique is a novel and promising magnetic resonance sequence; however, its application in nasopharyngeal carcinoma (NPC) lacks sufficient evaluation. This study aimed to assess the feasibility of the 3D CEST technique in predicting the short-term treatment outcomes for chemoradiotherapy (CRT) in NPC patients.

**Methods:**

Forty NPC patients and fourteen healthy volunteers were enrolled and underwent the pre-treatment 3D CEST magnetic resonance imaging and diffusion-weighted imaging (DWI). The reliability of 3D CEST was assessed in healthy volunteers by calculating the intra- and inter-observer correlation coefficient (ICC) for amide proton transfer weighted-signal intensity (APTw-SI) and magnetization transfer ratio (MTR) values. NPC patients were divided into residual and non-residual groups based on short-term treatment outcomes after CRT. Whole-tumor regions of interest (ROIs) were manually drawn to measure APTw-SI, MTR and apparent diffusion coefficient (ADC) values. Multivariate analysis and the receiver operating characteristic curve (ROC) were used to evaluate the prediction performance of clinical characteristics, APTw-SI, MTR, ADC values, and combined models in predicting short-term treatment outcomes in NPC patients.

**Results:**

For the healthy volunteer group, all APTw-SI and MTR values exhibited good to excellent intra- and inter-observer agreements (0.736–0.910, 0.895–0.981, all P > 0.05). For NPC patients, MTR values showed a significant difference between the non-residual and residual groups (31.24 ± 5.21% vs. 34.74 ± 1.54%, P = 0.003) while no significant differences were observed for APTw-SI and ADC values (P > 0.05). Moreover, the diagnostic power of MTR value was superior to APTw-SI (AUC: 0.818 vs. 0.521, P = 0.017) and comparable to ADC values (AUC: 0.818 vs. 0.649, P > 0.05) in predicting short-term treatment outcomes for NPC patients. The prediction performance did not improve even when combining MTR values with APTw-SI and/or ADC values (P > 0.05).

**Conclusions:**

The pre-treatment MTR value acquired through 3D CEST demonstrated superior predictive performance for short-term treatment outcomes compared to APTw-SI and ADC values in NPC patients after CRT.

**Supplementary Information:**

The online version contains supplementary material available at 10.1186/s40644-023-00602-6.

## Background

Nasopharyngeal carcinoma (NPC) patients have seen improved survival rate with the widespread application of intensity-modulated radiotherapy and optimized of chemotherapy strategies [1]. Despite these advancements, locoregional recurrence [2] and distant metastases following chemoradiotherapy (CRT) continue to significantly impact the survival and quality of life for approximately 10–15% and 15–30% of NPC patients, respectively [2, 3]. Therefore, an effective method to predict the short-term treatment outcome after CRT is crucial for both NPC patients and clinicians to make an informed decision. Accurate prediction of short-term therapeutic efficacy allows for pre-treatment adjustments, avoiding unnecessary treatments and toxic side effects, ultimately benefiting patients.

In clinical practice, the TNM staging system, based on morphological changes, falls short in adequately predicting therapeutic responses in NPC patients [4, 5]. Consequently, there is a need to explore approaches that detect functional changes in NPC, especially for promptly and effectively predicting therapeutic efficacy. Magnetic resonance imaging (MRI), including diffusion-weighted imaging (DWI) [6], intravoxel incoherent motion (IVIM) [7], and dynamic contrast-enhanced magnetic resonance imaging (DCE-MRI) [8], has increasingly played a vital role in evaluating short-term treatment outcomes in NPC patients. IVIM can efficiently reflect tumor responses to fractional radiotherapy and predict the radio-sensitivity of NPCs [9]. Reduced field of view (rFOV) DWI has been shown to improve diagnostic accuracy of tumors in the head and neck regions [10]. Additionally, multi-modality imaging biomarkers, such as the extracellular volume fraction (V_e_) from pre-treatment DCE-MRI and total lesion glycolysis (TLG) obtained by ^18^ F-FDG PET/CT, have been utilized to predict survival odds in patients with advanced NPC [11]. Furthermore, post-treatment mean kurtosis (K_mean_) derived from diffusion kurtosis imaging (DKI) has been identified as an independent predictor of radiotherapy response in NPC patients [12]. However, some results from these techniques have been contradictory [13, 14], necessitating the search for new imaging biomarkers to prognosticate therapeutic outcomes in NPC patients.

Magnetization transfer (MT) contrast is based on the interaction between semi-solid macromolecular protons and the free water protons of tissue [15]. Conventional magnetization transfer ratio (MTR) imaging, using non-selective saturation pulses, can effectively detect semi-solid macromolecules in organisms [16]. Chemical exchange saturation transfer (CEST) MRI, sensitive to certain chemical compounds with mobile molecules (e.g., NH, NH2 or -OH) in tissues, allows for quantification of endogenous mobile proteins and peptide metabolites in the millimolar range through selective saturation of solute protons at specific spectral frequencies with low-bandwidth radiofrequency (RF) irradiation [16–19]. Moreover, Amide proton transfer (APT) and Magnetization transfer (MT) maps could be obtained using 3D FSE-based CEST imaging, further enabling the measurement of APT weighted-signal intensity (APTw-SI) and MTR values. APTw-SI value serves as a vital biomarker of tumor proliferation and differentiation, while the MTR value can detect semi-solid macromolecules, such as bound proteins, lipids, carbohydrates, nucleic acid, membranes, and myelin in an organism [16, 20]. Presently, only two studies have reported on the potential of two-dimensional (2D) APTw-SI as a reliable biomarker in predicting the survival of NPC patients [2, 21]. However, the application of two-dimensional (2D) echo planar imaging CEST sequence has limitations such as structural distortion from image acquisition-induced susceptibility to field inhomogeneity, especially in cavities and air-tissue interfaces.

The volume-excited three-dimensional (3D) fast spin echo CEST sequence possesses features of a relatively large scan coverage for the entire structures of interest, higher signal-to-noise ratio (SNR), and reduced image distortion, as has been reported in glioma [22], rectal adenocarcinoma [23] and bladder cancer [24]. Therefore, this study aims to systematically evaluate the reliability of the 3D CEST sequence in predicting the short-term outcomes for NPC patients with APTw-SI, MTR and ADC values, and even their combined models after CRT.

## Methods

### Patients and volunteers

**Fig. 1:**
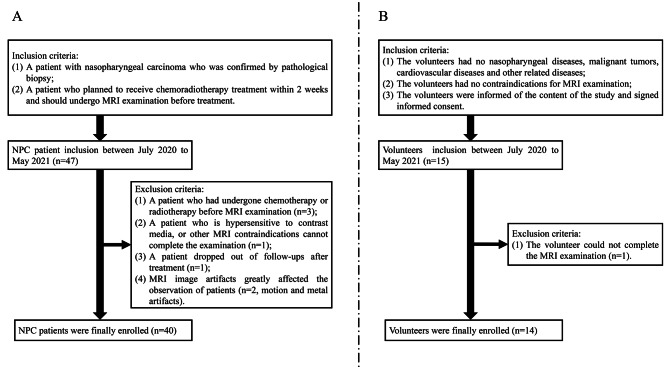
The NPC patients and healthy volunteers’ inclusion flow chart

This prospective study was approved by the hospital ethics committee. The authors confirmed that all data were collected after the participants’ completion of written informed consent. All methodologies adhered rigorously to the applicable guidelines, and regulations, and the principles outlined in the Declaration of Helsinki. Participant enrollment occurred from July 2020 to May 2021. And the inclusion flow chart for both NPC patients and healthy volunteers can be found in Fig. 1.

Ultimately, our study identified a total of 40 patients (mean age: 51.2 ± 12.6 years, age range: 14–80 years). Additionally, 14 healthy volunteers (mean age: 52.2 ± 10.3 years; age range: 27–74 years) were included to assess the reliability of the 3D CEST MRI sequence.

### Clinicopathological characteristics

Patients specific information encompassing age, gender, tumor diameter (measured at the largest solid tumor slice), T stage, N stage, AJCC/UICC stage, histology, and the radiological status of invasion to the skull base were meticulously gathered. Staging of nasopharyngeal carcinoma adhered to the 8th edition of the American Joint Committee on Cancer (AJCC stage)/the Union for International Cancer Control (UICC) TNM Classification, employing pre-treatment MRI images as the basis [25, 26]. Furthermore, based on the histology type, the tumors were categorized into differentiated non-keratinizing (DNK) and undifferentiated non-keratinizing (UNK). The radiological status of tumor invasion into the skull base was executed through a comprehensive evaluation of radiological and clinical findings.

### MRI examination

All NPC patients underwent MR examination with a 3D CEST, reduced field-of-view (rFOV) diffusion weighted imaging (DWI) and routine MR sequences on 3.0 T MR scanners (Discovery MR750w, GE Healthcare, USA) using 16-channel flex large array coil and 6-channel flex array coil due to the use of a fixed device of the radiotherapy positioning body membrane. The rFOV-DWI and 3D CEST imaging were performed prior to contrast agent administration, and detailed parameters can be found in **Supplementary Table 1**.

Oblique axial 3D CEST images were acquired using a FSE sequence combined with chemical shift–selective (CHESS) fat suppression to optimize the signal-to-noise ratio (SNR). A pseudo continuous pulse was applied with a total duration of 3 s that comprises pulse duration of 0.232 ms and a pulse interval of 0.56 ms and a saturation power level of 1.5 mT for seven different saturation frequency offsets (± 3.0, ± 3.5, ± 4.0, and 7.0 ppm). The first 6 points were used to compute the resulting magnetization transfer ratio asymmetry (MTR_asym_) and further obtain APTw-SI, while the last point was used to calculate the magnetization transfer ratio (MTR). The water signal saturation is measured as a function of saturation frequency and the water frequency (around 4.75 ppm in the proton MR spectrum) and placed at 0 ppm in the Z-spectrum. A B0 field was compensated with phase images acquired with an iterative decomposition of water and fat with echo asymmetry and least-squares estimation quantitation sequence (IDEAL IQ). Additionally, the ZIP2 technique was also used in 3D CSET sequence for obtaining 4 mm thickness of volume image with an interpolation reconstruction. For healthy volunteers, solely 3D CEST was scanned twice using identical imaging parameters. In enrolled NPC patients, oblique axial 3D CEST encompassing the entire tumor to the greatest extent possible was acquired.

### Data analysis

#### 3D CEST post-processing

All 3D CEST data underwent automated post-processed to generate the APT and MT weighted images, accomplished via a published algorithm [17], followed by transfer to the Advanced Workstation 4.7 (GE Healthcare, Waukesha, USA ). APTw-SI was measured from APT weighted images computed as the magnetization transfer ratio asymmetry (MTR_asym_) at 3.5 ppm:

1 $${\rm{APTw}} - {\rm{SI}} = MT{R_{asym}}\left( {3.5ppm} \right) = \frac{{{S_{ - 3.5ppm}} - {S_{3.5ppm}}}}{{{S_0}}}$$

Here, S_− 3.5ppm_ and S_3.5ppm_ represent signal intensities at − 3.5 and 3.5 ppm, respectively, and S_0_ denotes the unsaturated signal intensity.

Furthermore, MTR was computed from the MT images utilizing the formula:

2 $$MTR\left( {7.0ppm} \right) = 1 - \frac{{{S_{7.0ppm}}}}{{{S_0}}}$$

**Fig. 2:**
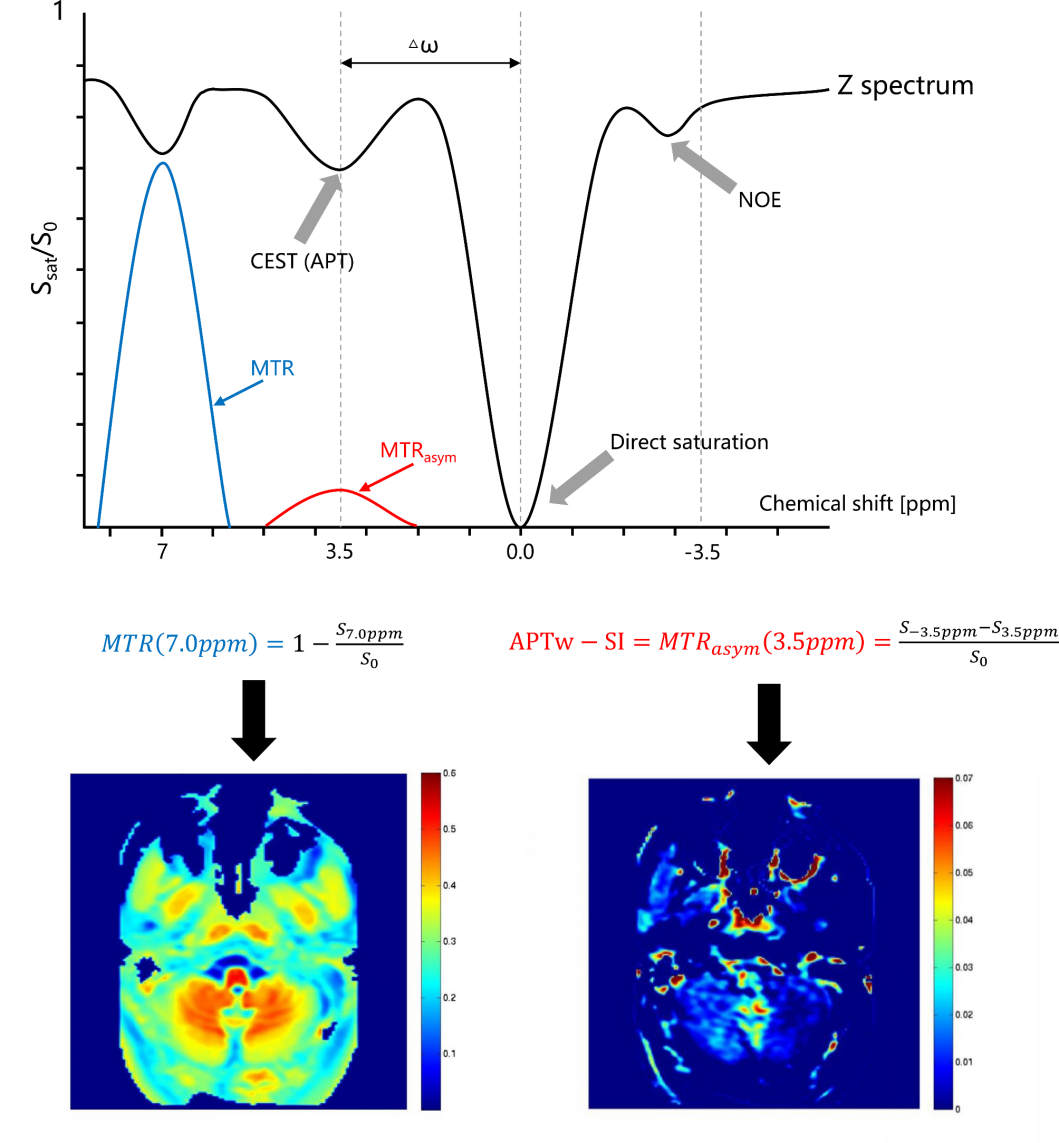
The principle of the 3D CEST pulse sequence. By applying a selective saturation pulse for amide proton at 3.5 ppm and non-selective saturation pulse at 7.0 ppm to achieve proton exchange with bulk water, the values of APTw-SI: MTR_asym_ (3.5ppm) and MTR (7.0ppm) can be calculated according to the above equation and displayed in colors

Where S_7.0 ppm_ and S_0_ signify signal intensities at 7.0 ppm with and without magnetic transfer, respectively (Fig. 2) [17, 19, 27, 28].

#### CEST measurements

**Fig. 3:**
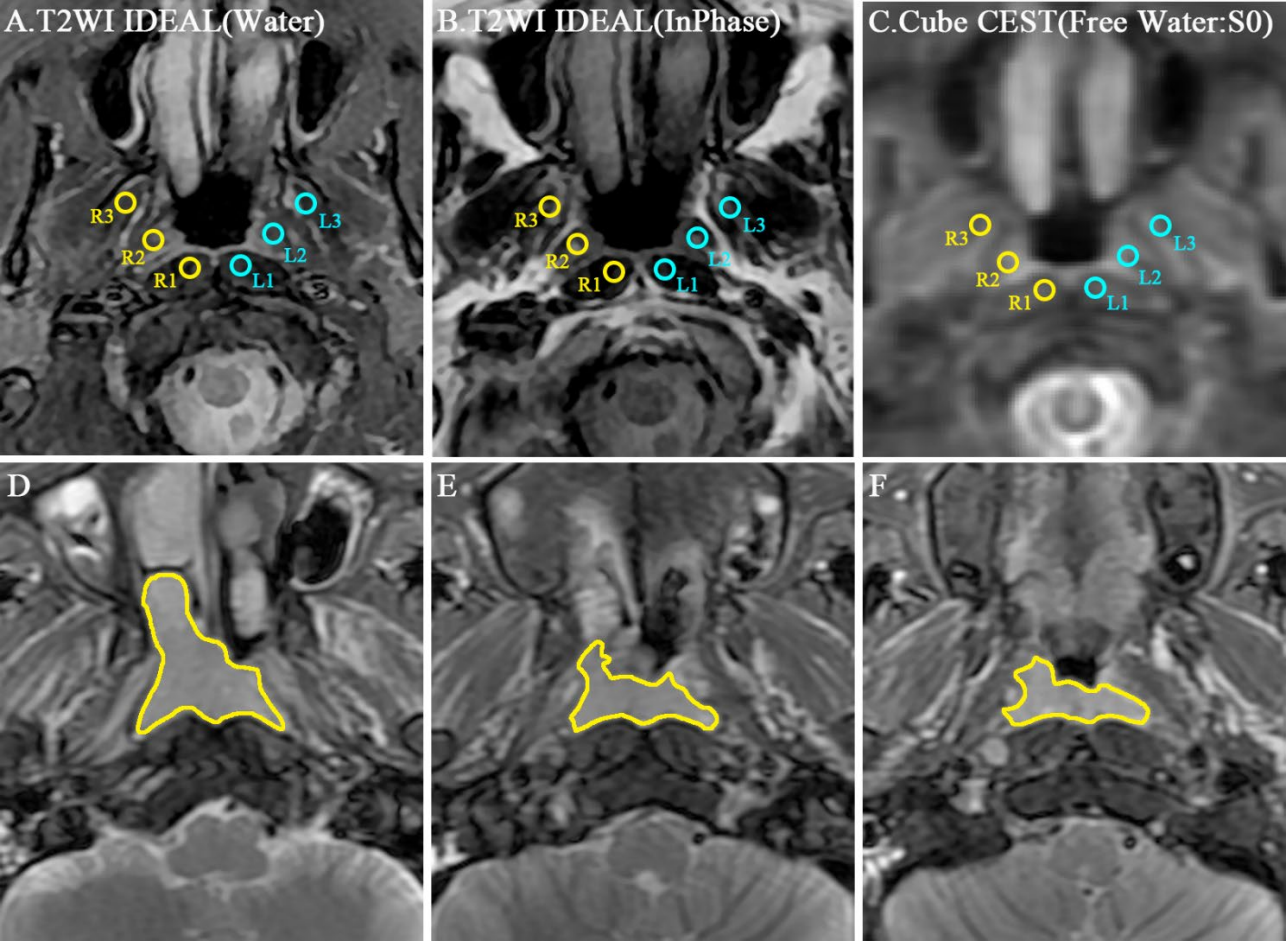
Illustrated ROI positions. **A-C**: The repeatability of 3D CEST was evaluated in 6 ROIs of the nasopharynx (R1: right longus capitis, R2: right tensor veli palatini, R3: right medial pterygoid, L1: left longus capitis, L2: left tensor veli palatini, L3: left medial pterygoid). **D-F**: An example of whole tumor ROI sketched at the margin of the whole primary tumor for all sections on the same patient images

In the case of healthy volunteers, regions of interest (ROIs) were manually delineated on an axial T2WI images, which were subsequently projected onto the APT and MT maps to obtain mean APTw-SI and MTR values independently and double-blindly by two radiologists (Reader 1 and Reader 2, with 5 and 3 years of clinical imaging diagnosis experience, respectively). If there have motion or distortion in the image, the ROI is manually corrected. Six rounds ROIs (each around 20 mm^2^) were delineated on the nasopharynx, including bilateral longus capitis, tensor veli palatini, medial pterygoid (R1, R2, R3, L1, L2, L3 respectively). In addition, Reader 1 repeated this step for intra-observer analysis after a two-week interval (Fig. 3A-C).

For the NPC patients, a whole-tumor ROI was drawn on primary tumor based on axial T2WI images [29, 30], which was then extended to the APT, MT and ADC maps for determination of mean APTw-SI, MTR, and ADC values respectively. These whole-tumor ROIs were outlined along the primary tumor margin across all slices (Fig. 3D-F). The delineation of the tumor margin in axial T2WI images and CEST sequences were confirmed by the consensus of two radiologists (Reader 1 and Reader 2), who were blinded to the patients’ therapy response.

### Follow-ups

**Fig. 4:**
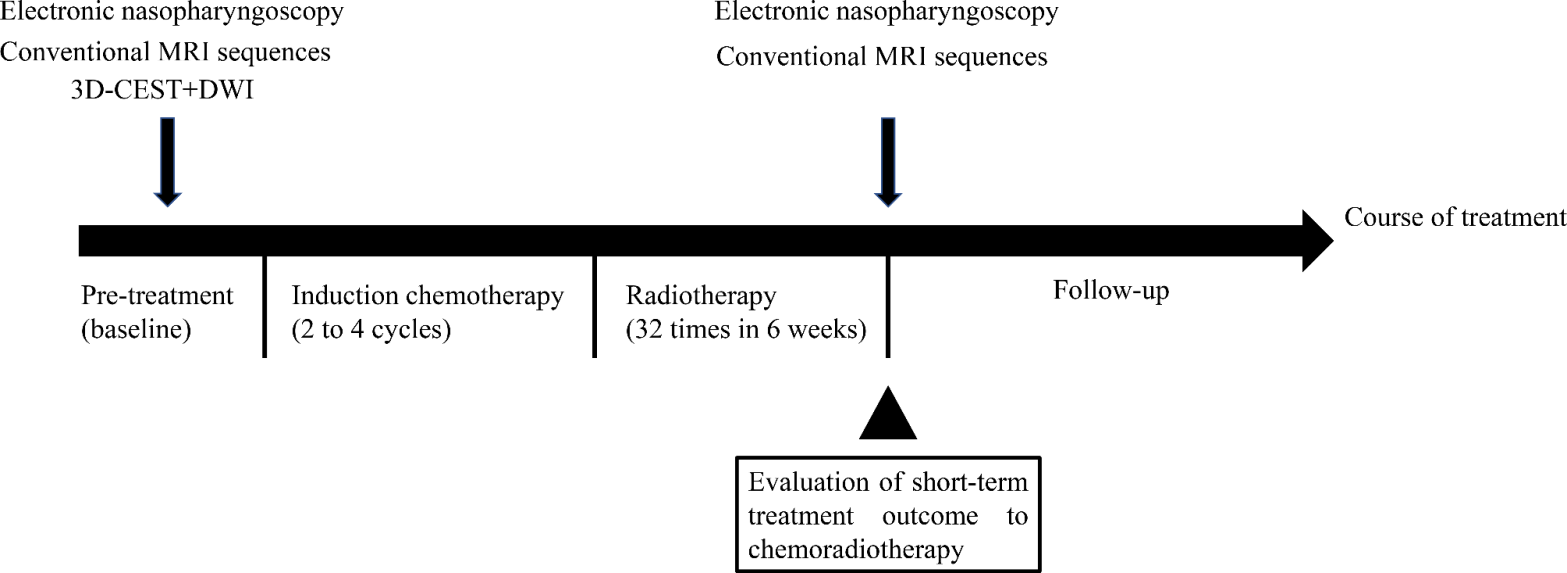
Schematic diagram of treatment course, MRI examinations, electronic nasopharyngoscopy, outcome evaluation and follow-ups for NPC patients. CEST: Chemical Exchange Saturation Transfer; DWI: Diffusion weighted imaging; CRT: Chemoradiotherapy

Each NPC patient underwent 2 to 4 cycles of induction chemotherapy, using the GP regimen (gemcitabine + cisplatin). Subsequently, radiotherapy involving 6MV-X-ray and intensity-modulated radiotherapy (IMRT) were administered at a dose of 2.2 Gy per session, totaling 32 sessions over a span of 6 weeks. MRI examinations and electronic nasopharyngoscopy were performed before treatment and post radiotherapy (Fig. 4).

The short-term therapeutic outcomes following CRT was evaluated by two senior radiologists (Reader 3 and Reader 4, with 15 and 20 years of clinical imaging diagnosis experience, respectively). These radiologists were kept unaware of clinical data, MRI images, electronic nasopharyngoscopy and pathology information. The maximum tumor diameter was measured on pre- and post-CRT axial T2W and T1W enhanced images. Treatment outcomes were categorized into the four groups (complete response, CR; partial response, PR; progressive disease, PD and stable disease, SD) based on the Response Evaluation Criteria in Solid Tumors (RECIST) 1.1 version guidelines [31]. Specifically, CR indicated no evidence of residual primary tumor and metastatic lymphadenopathy on MR imaging and pharyngorhinoscopy, with a regression ratio of 100%; PR implied evidence of residual primary tumor or metastatic lymphadenopathy, with a regression ratio exceeding 30%; PD signified disease progress, with a progression ratio surpassing 20%; SD stood between PR and PD, with a regression ratio blow 30% and progression ratio under 20%. Subsequently, the short-term treatment outcomes were dichotomized into the residual group (comprising NPC patients with PR, SD and PD) and the non-residual group (encompassing NPC patients with CR) [32].

### Statistical analysis

For healthy volunteers image data, the intraclass correlation coefficient (ICC) was used to examine intra-observer and inter-observer agreements of APTw-SI and MTR values. ICC values ranging from 0.60 to 0.80 and from 0.80 to 1.0 indicated good and excellent agreements for APTw-SI and MTR values, respectively. Bland-Altman plots were employed to illustrate the reliability of APTw-SI and MTR values measurements [33]. The mean absolute difference (bias) and the 95% confidence interval (95%CI) of the mean difference (limits of agreement, LOA) between the first and second APTw-SI and MTR values were also presented [33, 34].

For NPC patient image data, independent sample T-test or Mann-Whitney U test were used to evaluate difference in continuous variables (age, diameter, APTw-SI, MTR and ADC) between groups with distinct short-term treatment outcomes. The Chi-square test was applied for analyzing differences in discrete variables such as gender, T stage, N stage, AJCC/UICC stage, histology, and the radiological status of skull base invasion. Multivariate analysis and receiver operating characteristic curve (ROC) were used to evaluate the prognostic efficacy of APTw-SI, MTR and ADC values in short-term treatment outcomes. This involved calculating the area under the curve (AUC), 95% confidence interval (95% CI), sensitivity, and specificity for their predictive performance. Furthermore, the prognostic efficacy of combined models involving APTw-SI, MTR and/or ADC (APTw-SI + ADC model, MTR + APTw-SI model, MTR + ADC model, and MTR + APTw-SI + ADC model) in predicting the short-term treatment outcomes of NPC patients post CRT were evaluated.

SPSS software (version 29.0, Chicago, IL, USA) was employed for statistical analysis and ROC curve generation. A significance level of P value < 0.05 was applied to determine statistically significant differences. Bland-Altman plots were constructed using MedCalc statistical software version 20 (MedCalc Software Ltd, Ostend, Belgium).

## Results

### Reliability analysis of CEST acquirement

**Fig. 5:**
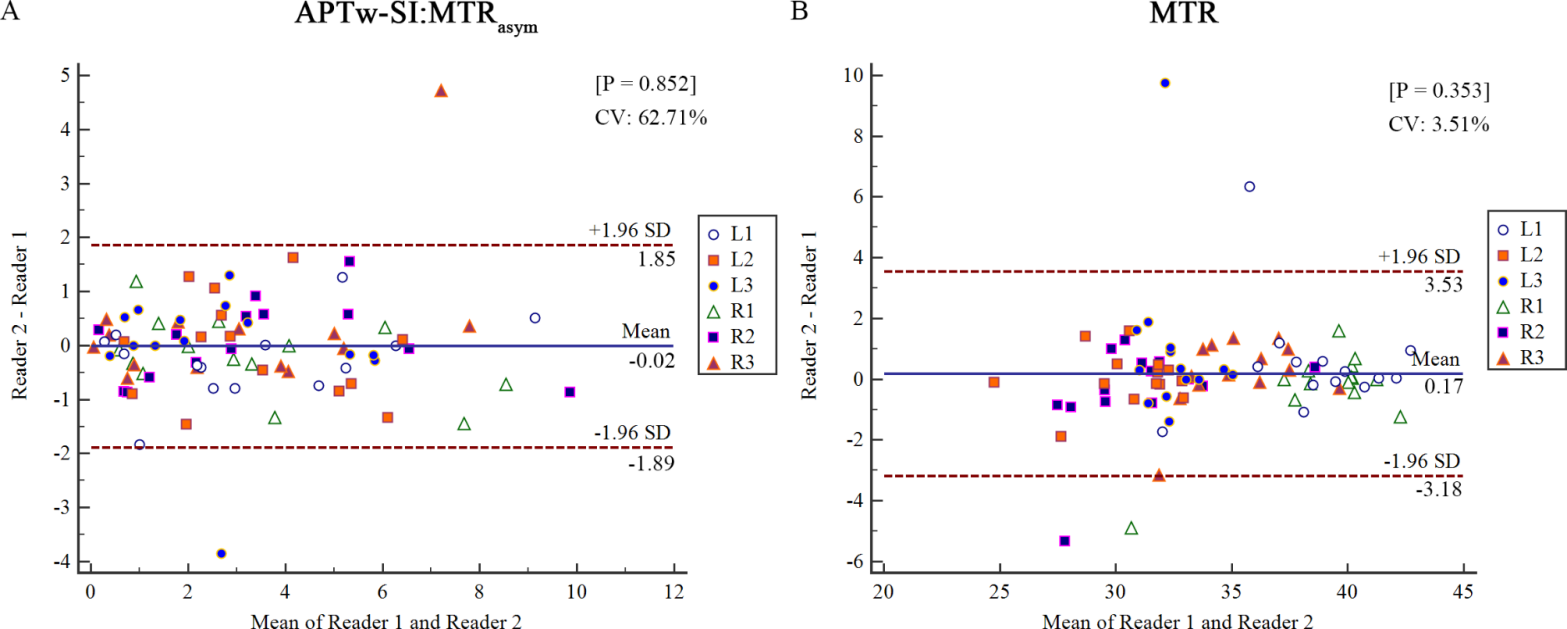
Bland–Altman plots for the repeatability of APTw-SI **(A)** and MTR **(B)** measurements. Plots include the 95% limits of agreement (LOA), mean absolute difference and coefficient of variation (CV). Most dots were located within the 95% LOA. Only 2 points (2/84, 2.4%) in (A) and only 4 points (4/84, 4.8%) in (B) exceed the 95% LOA range for APTw-SI and MTR measurements, respectively. Blue line = mean absolute difference, red dotted lines = 95% confidence interval of the mean difference (LOA). R1 = right longus capitis, R2 = right tensor veli palatini, R3 = right medial pterygoid, L1 = left longus capitis, L2 = left tensor veli palatini, L3 = left medial pterygoid

The APTw-SI and MTR values showed good and excellent intra- and inter-observer agreements and showed no significant inter-measurement difference (APTw-SI: 0.821–0.910 and 0.899–0.981; MTR: 0.736–0.867 and 0.895–0.965; all P > 0.05) for 6 ROIs in the healthy volunteers (Table 1). For a comprehensive depiction of the mean absolute difference, 95% limits of agreement (LOA), and coefficient of variation (CV) between reader 1 and reader 2 for measured MTR and APTw-SI values, refer to Fig. 5.

**Table 1:** The measurements of APTw-SI and MTR in six representative positions and intra- and inter-observer agreements

		Reader 1			Reader 2		Agreement	
		First measurements	Second measurements	P Value	First measurements	P Value	Intra-observer	Inter-observer
APTw-SI: MTR_asym_ (%)	R1	3.369 ± 2.742 (1.786–4.952)	3.424 ± 2.335 (2.076–4.772)	0.908	3.180 ± 2.399 (1.795–4.565)	0.330	0.862	0.981
	R2	3.309 ± 2.653 (1.778–4.841)	2.652 ± 1.901 (1.555–3.750)	0.123	3.383 ± 2.748 (1.796–4.970)	0.712	0.883	0.981
	R3	2.881 ± 2.327 (1.537–4.224)	2.622 ± 1.821 (1.571–3.673)	0.435	3.199 ± 2.941 (1.501–4.898)	0.384	0.910	0.934
	L1	3.442 ± 2.430 (2.039–4.845)	3.019 ± 1.912 (1.915–4.123)	0.356	3.197 ± 2.704 (1.636–4.759)	0.224	0.833	0.980
	L2	3.345 ± 1.989 (2.196–4.494)	3.162 ± 2.053 (1.977–4.347)	0.598	3.302 ± 1.823 (2.250–4.355)	0.869	0.891	0.933
	L3	2.624 ± 2.032 (1.451–3.798)	2.314 ± 1.334 (1.543–3.084)	0.401	2.591 ± 1.892 (1.499–3.684)	0.919	0.821	0.899
MTR (%)	R1	38.884 ± 3.354 (36.947–40.820)	38.660 ± 4.032 (36.332–40.988)	0.801	39.194 ± 2.272 (37.882–40.506)	0.393	0.763	0.929
	R2	30.836 ± 3.300 (28.931–32.742)	30.189 ± 2.734 (28.611–31.768)	0.265	31.207 ± 2.650 (29.677–32.737)	0.400	0.867	0.924
	R3	35.332 ± 2.489 (33.895–36.769)	35.140 ± 2.014 (33.978–36.303)	0.547	34.443 ± 1.946 (33.319–35.567)	0.128	0.736	0.930
	L1	38.856 ± 2.913 (37.174–40.537)	38.999 ± 3.150 (37.180–40.818)	0.846	38.346 ± 3.049 (36.586–40.107)	0.319	0.753	0.895
	L2	30.400 ± 2.432 (28.996–31.804)	30.530 ± 2.608 (29.024–32.036)	0.822	30.491 ± 2.272 (29.179–31.802)	0.809	0.786	0.965
	L3	32.992 ± 1.684 (32.020–33.964)	32.944 ± 2.161 (31.697–34.192)	0.902	33.114 ± 1.997 (31.961–34.267)	0.612	0.842	0.940

**Note**: All values were shown in arithmetic mean ± standard deviation, and the 95% confidence interval for the mean was included in parentheses. APTw-SI: amide proton transfer weighted signal intensity; MTR: magnetization transfer ratio; MTR_asym_: the asymmetric magnetization transfer ratio; R1: right longus capitis, R2: right tensor veli palatini, R3: right medial pterygoid, L1: left longus capitis, L2: left tensor veli palatini, L3: left medial pterygoid. P > 0.05 was considered not statistically significant.

**Table 2:** Clinicopathological characteristics of patients with nasopharyngeal carcinoma

		CRT		P value
		Non-residual(n = 28)	Residual(n = 12)	
Age (mean ± SD, years)		53.04 ± 12.84	46.92 ± 11.20	0.160
Gender	Male(n = 31)	21(67.7%)	10(32.3%)	0.447
	Female(n = 9)	7(77.8%)	2(22.2%)	
Diameter(mm)		30.35 ± 10.31	33.63 ± 8.28	0.337
T stage	T1(n = 3)	3(100%)	0	0.623
	T2(n = 3)	2(66.7%)	1(33.3%)	
	T3(n = 21)	15(71.4%)	6(28.6%)	
	T4(n = 13)	8(61.5%)	5(38.5%)	
N stage	N0(n = 5)	3(60.0%)	2(40.0%)	0.764
	N1(n = 12)	9(75.0%)	3(25.0%)	
	N2(n = 13)	10(76.9%)	3(23.1%)	
	N3(n = 10)	6(60.0%)	4(40.0%)	
AJCC/UICC stage	II(n = 2)	2(100%)	0	0.370
	III(n = 14)	11(78.6%)	3(21.4%)	
	IV(n = 24)	15(62.5%)	9(37.5%)	
Histology	DNK(n = 15)	13(86.7%)	2(13.3%)	0.074
	UNK(n = 25)	15(60.0%)	10(40.0%)	
Invasion of the skull base	Yes (n = 23)	15(65.2%)	8(34.8%)	0.431
	No (n = 17)	13(76.5%)	4(23.5%)	

**Note**: CRT: chemoradiotherapy; AJCC/UICC stage: Nasopharyngeal carcinoma staging was performed according to the 8th edition of the American Joint Committee on Cancer (AJCC stage)/the Union for International Cancer Control (UICC) TNM Classification based on pre-treatment MRI images. DNK: Differentiated non-keratinizing; UNK: Undifferentiated non-keratinizing. P < 0.05 was considered statistically significant.

### Clinicopathological characteristics of NPC patients

Following chemoradiotherapy, 28 of 40 NPC patients fell into the non-residual group (complete response), while the remaining 12 were classified within the residual group (not complete response). A thorough analysis of the non-residual and residual groups unveiled no statistically significant differences across various parameters including age, gender, diameter, T stage, N stage, AJCC/UICC stage, histology and the radiological status of invasion to the skull base (all P > 0.05; Table 2).

### Prediction performance of APTw-SI, MTR, ADC values and their combined models for short-term treatment outcome

There was no significant difference in APTw-SI and ADC values between non-residual and residual groups (all P > 0.05) (Table 3). However, a notable discrepancy emerged in MTR values between the non-residual and residual groups (31.24 ± 5.21% vs. 34.74 ± 1.54%, P = 0.003).

**Table 3:** The findings of APTw-SI, MTR and ADC values for predicting the short-term treatment outcome in NPC patients after chemoradiotherapy

	CRT		P value
	Non-residual(n = 28)	Residual(n = 12)	
APTw-SI: MTR_asym_ (%)	1.72 ± 1.21	1.64 ± 1.22	0.854
MTR (%)	31.24 ± 5.21	34.74 ± 1.54	0.003**
ADC value (10^− 6^ mm^2^/sec)	1004.34 ± 281.71	912.54 ± 175.29	0.304

**Note**: All values were shown in mean ± standard deviation. CRT: chemoradiotherapy; APTw-SI: amide proton transfer weighted signal intensity; MTR: magnetization transfer ratio; MTR_asym_: the asymmetric magnetization transfer ratio; ADC: apparent diffusion coefficient. *: *P*<0.05, **: *P*<0.01.

**Fig. 6:**
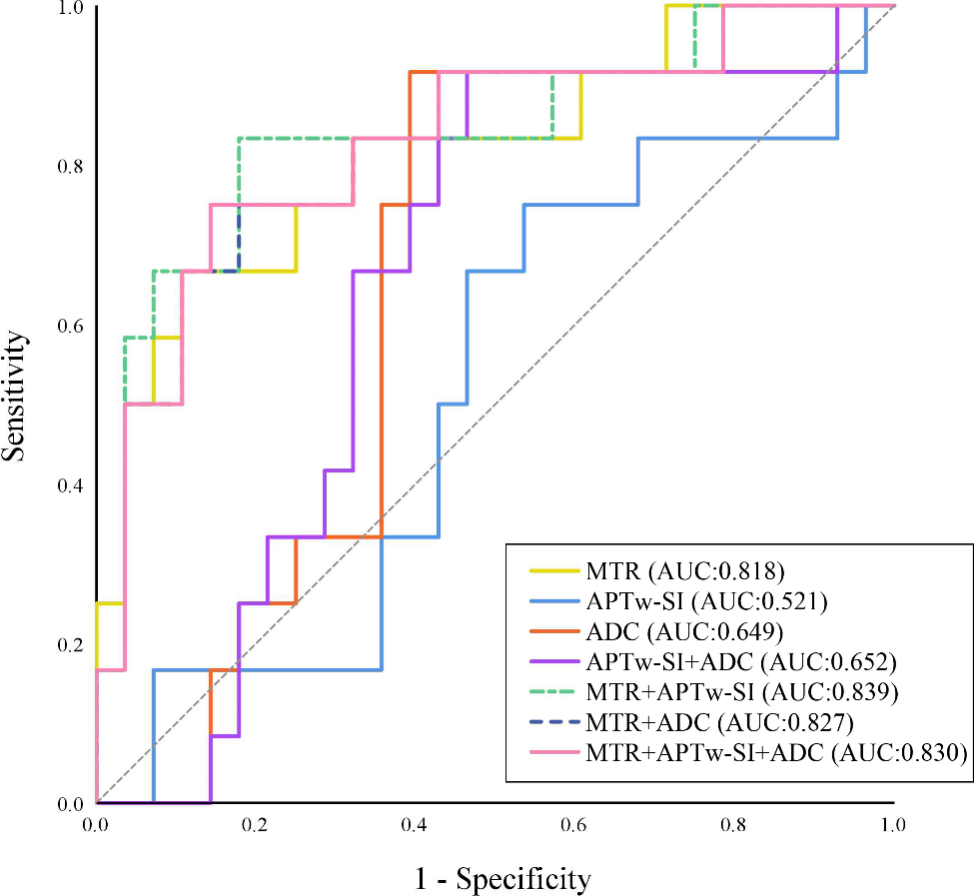
ROC curves for multivariate analysis of APTw-SI, MTR and ADC values in predicting the short-term treatment outcome for NPC patients after CRT. The MTR value had a higher diagnostic power in distinguishing the non-residual from residual groups for NPC patients (AUC:0.818). The difference in AUC between MTR and APTw-SI model were statistically significant (AUC:0.818 vs. 0.521, P = 0.017). However, there were no significant difference between MTR value and other four multivariate prediction models (all P > 0.05)

Remarkably, the predictive capacity of the MTR value proved significantly higher [AUC: 0.818 (0.665–0.922)], establishing a cutoff value of 34.66, (*P*<0.001) for distinguishing between non-residual and residual groups. Visual representations of the ROC curves for MTR, APTw-SI and ADC values in predicting the short-term therapeutic outcome for NPC patients post CRT are showcased in Fig. 6. Worth mentioning is the statistically significant difference between the AUCs of the MTR and APTw-SI value models (AUC:0.818 vs. 0.521, P = 0.017).

**Fig. 7:**
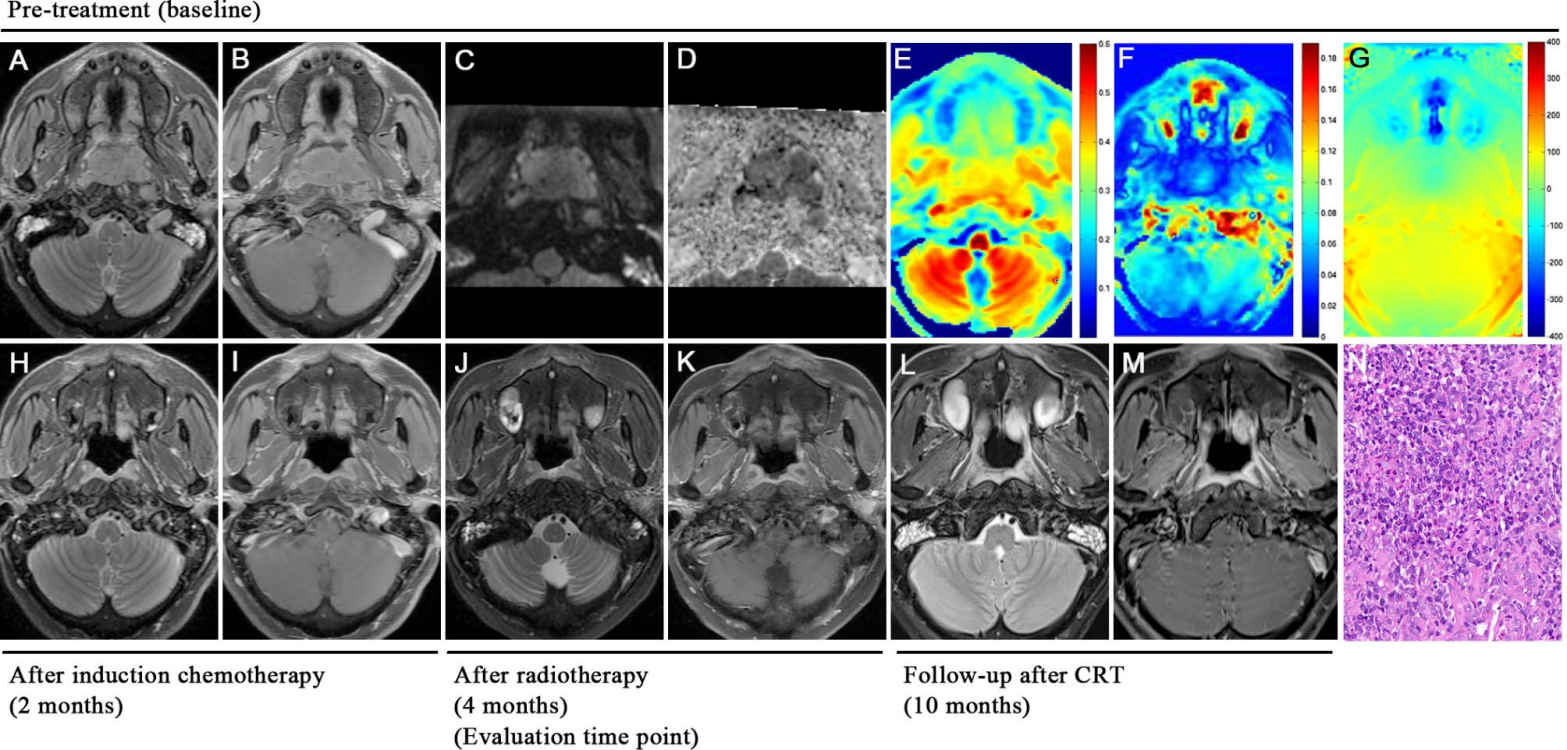
A 42-year-old male patient with nasopharyngeal carcinoma presented with complete response (non-residual lesions) after chemoradiotherapy. T2WI sequence **(A)**, T1WI contrast enhancement sequence **(B)**, rFOV DWI sequence **(C)**, and ADC map **(D)** showed the location of huge tumors in the nasopharynx. MTR (7.0ppm) color maps **(E)** and APTw-SI: MTR_asym_ (3.5ppm) color maps **(F)** showed that the tumor is of equal or slightly low signal and of slightly low signal, respectively. Based on B0 map **(G)**, tumor ROI showed homogenous and reasonable phase difference (< 60 Hz) compared to nasal cavity. MRI examination at the end of induction chemotherapy showed that the tumor had nearly disappeared **(H, I)**. MRI examinations and electronic nasopharyngoscopy at the end of chemoradiotherapy showed that the tumor had completely disappeared (complete response, CR) and was classified into the non-residual group **(J, K)**. MRI examinations at 6 months **(L, M)** after the last chemoradiotherapy treatment showed no local recurrence of the lesions and it verifies the previous judgment. Hematoxylin-eosin staining of pathological biopsy showed differentiated nonkeratinizing nasopharyngeal carcinoma **(N)**

**Fig. 8:**
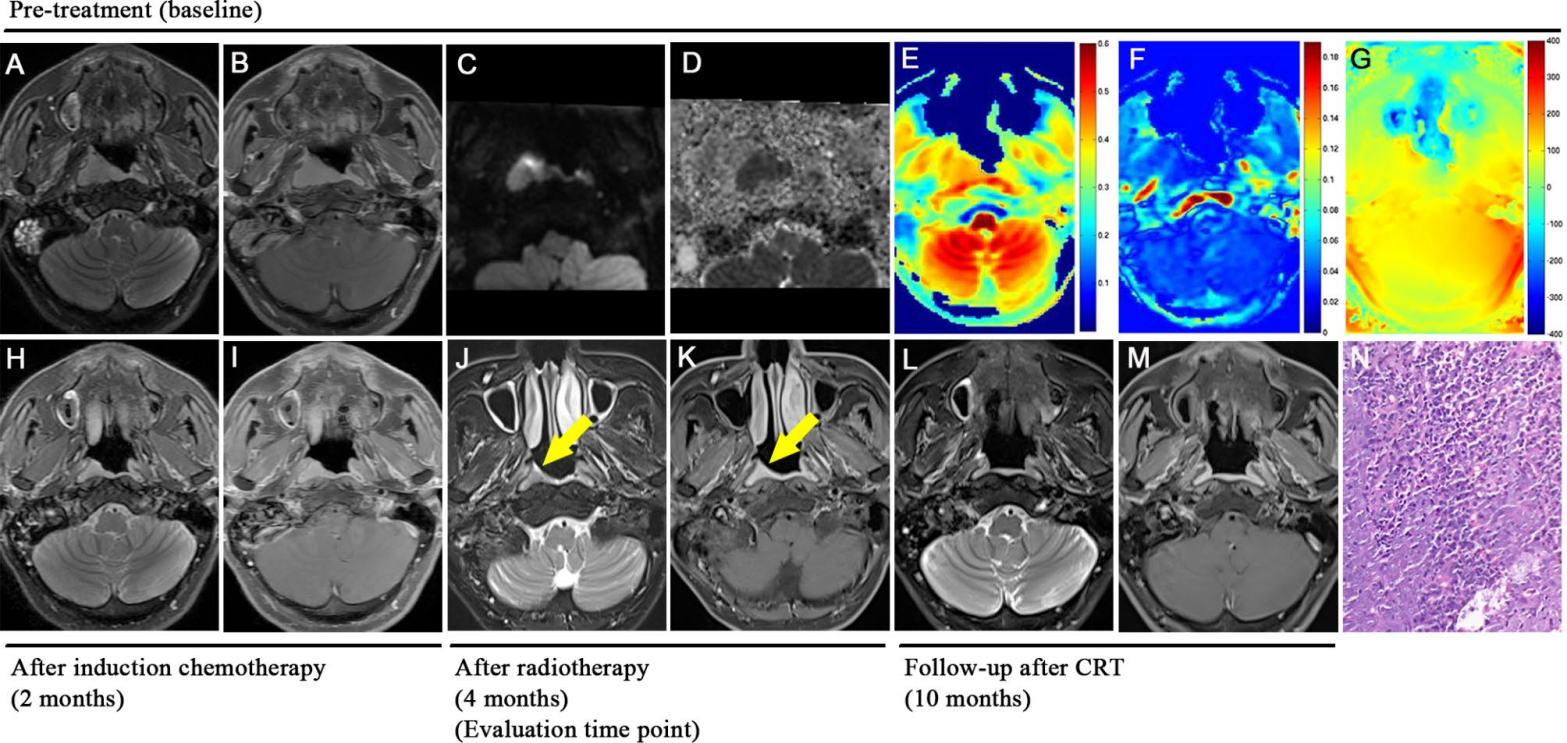
A 47-year-old male patient with nasopharyngeal carcinoma presented with residual lesions after chemoradiotherapy. T2WI sequence **(A)**, T1WI contrast enhancement sequence **(B)**, rFOV DWI sequence **(C)**, and ADC map **(D)** showed the location of huge tumors in the right nasopharynx. MTR (7.0ppm) color maps **(E)** and APTw-SI:MTR_asym_ (3.5ppm) color maps **(F)** showed that the tumor is of equal or high signal and of slightly low signal, respectively. Based on B0 map **(G)**, tumor ROI showed homogenous and reasonable phase difference (< 60 Hz) compared to nasal cavity. MRI examination at the end of induction chemotherapy showed that the tumor had regression significantly **(H, I)**. MRI examinations and electronic nasopharyngoscopy at the end of chemoradiotherapy showed that the tumor had small residual lesions (partial response, PR) (yellow arrow) and was classified into the residual group **(J, K)**. MRI examinations at 6 months **(L, M)** after the last chemoradiotherapy treatment showed the tumor had completely disappeared and it verifies the previous judgment. Hematoxylin-eosin staining of pathological biopsy showed differentiated nonkeratinizing nasopharyngeal carcinoma **(N)**

However, among the four multivariate prediction models [APTw-SI + ADC model: (AUC:0.652), MTR + APTw-SI model: (AUC:0.839), MTR + ADC model: (AUC:0.827) and MTR + APTw-SI + ADC model: (AUC:0.830)], there was no significant distinction between the MTR value and any of the models (all P > 0.05) (Table 4; Figs. 6, 7 and 8).

**Table 4:** The multivariate analysis for prediction performance of APTw-SI, MTR and ADC values in the short-term treatment outcome for NPC patients after chemoradiotherapy

Parameters	AUC (95% CI)	Sensitivity (95% CI)	Specificity (95% CI)	P value
MTR	0.818(0.665–0.922)	89.29(71.8–97.7)	66.67(34.9–90.1)	<0.001**
APTw-SI: MTR_asym_	0.521(0.357–0.681)	75.00(42.8–94.5)	46.43(27.5–66.1)	0.839
ADC	0.649(0.482–0.793)	91.67(61.5–99.8)	60.71(40.6–78.5)	0.109
APTw-SI + ADC	0.652(0.485–0.795)	53.57(33.9–72.5)	91.67(61.5–99.8)	0.099
MTR + APTw-SI	0.839(0.689–0.936)	82.14(63.1–93.9)	83.33(51.6–97.9)	<0.001**
MTR + ADC	0.827(0.675–0.928)	82.14(63.1–93.9)	75.00(42.8–94.5)	<0.001**
MTR + APTw-SI + ADC	0.830(0.678–0.930)	85.71(67.3–96.0)	75.00(42.8–94.5)	<0.001**

**Note**: AUC: area under the ROC curve; 95%CI: 95% Confidence interval; APTw-SI: amide proton transfer weighted signal intensity; MTR: magnetization transfer ratio; MTR_asym_: the asymmetric magnetization transfer ratio; ADC: apparent diffusion coefficient. *: *P*<0.05,**: *P*<0.01.

## Discussion

Our study firstly and systematically reported the good to excellent repeatability of APTw-SI and MTR values using the 3D CEST technique in healthy volunteers. Additionally, pre-treatment MTR values exhibited good predictive performance for short-term treatment outcomes in NPC patients after CRT.

Pre-treatment mean APTw-SI value was higher in non-residual groups than the residual groups, which aligned with previous findings of a higher mean APTw-SI values in chemoradiotherapy-responding NPC patients, particularly within a ROI on the two-dimensional APT map with the maximum cross-section lesion, compared to non-responders [21]. Despite the tendency, the pre-treatment mean APTw-SI value failed to predict short-term treatment outcomes [21], early response to induction chemotherapy [35], and 2-year long-term locoregional relapse-free survival [2]. Earlier studies have shown that APTw-SI values gradually decrease during the course of treatment, and APTw-SI values can show pre- and post-treatment differences earlier than changes in the volume of NPC tumors [5]. Therefore, the pre-treatment APTw-SI value exhibited limited capability in predicting the short-term treatment outcomes for NPC patients after CRT. Importantly, concerns about susceptibility artifacts at the junction of the nasal bone and air affecting the APTw-SI value at 3.5 ppm were addressed since the drawn ROI on the B0 map showed homogenous and reasonable phase differences (< 60 Hz) compared to the nasal cavity (-122.1 ± 23.5 Hz) (Figs. 7 and 8) [36–38]. Additionally, the mean APTw-SI value is ≥ 0 indicates that the APT signal dominates and the nuclear overhauser enhancement (NOE) effect is minimized when the saturation power is higher than 1uT [39]. That is, these findings eliminate susceptibility-caused alterations in APTw-SI value. As a result, APTw-SI values at multiple timepoints may more effectively provide dynamic protein change throughout the treatment course than a stationary baseline pre-treatment status for predicting the treatment response of NPC patients.

In contrast, our study indicates that NPC patients with lower MTR value demonstrated better treatment outcome, as the pre-treatment MTR value showed a higher prediction efficacy in determining the short-term treatment outcome of NPC patients after CRT (*P*<0.01). A non-selective saturation pulse in MT imaging can cause the interaction between semi-solid macromolecular protons (bound water pool) and free water protons in the tissues [19]. In other words, MT imaging technique can detect macromolecules, such as bound proteins, lipids, carbohydrates, nucleic acid, membranes and myeline in an organism [16, 20]. The MTR value reflects the amount and complexity of structural macromolecules [40], immobile macromolecules in tissues [41] and anatomical tissue destruction in details while APT is not sufficiently sensitive to structures with relatively low concentrations of mobile proteins and peptides [42]. MT technology has been applied to the brain [43], intestine [44] and kidney [19, 45] studies, research on nasopharyngeal carcinoma, especially in relation to chemoradiotherapy response prediction, remains scarce though. Interestingly, previous findings aligned with our results. For instance, Mehrabian H et al. [46] observed significant MT contrast differences between radiation necrosis and tumor progression in brain metastases patients. Additionally, Martens MH et al. [47] found that the mean MTR value of fibrosis was 37.7%, which was significantly higher than that of the residual tumor (29.6%), the normal rectal wall (30.2%), and the edematous rectal wall (18.2%) in patients with locally advanced rectal cancer treated with CRT. In head and neck tumors, Takashima S et al. [48] reported a significant positive correlation between MTR value and cell proliferative fractions and the number of macromolecular proteins in the nuclei based on the result of flow cytometry that can rapidly, objectively, and quantitatively measure DNA content of the nuclei in a lesion. Therefore, intermediate filament proteins other than macromolecular proteins in the nuclei and cytoplasm may influence the MTR of parotid lesions to some extent [48]. The above findings supported out results. We speculated that a lower MTR values for nasopharyngeal carcinoma, may indicate a lower cell proliferation fraction (i.e., a lower degree of malignancy) and it ultimately leading to better treatment outcomes after CRT. Therefore, our research provides evidence that lower MTR values before chemoradiotherapy could serve as a marker of favorable outcomes for NPC patients.

While diffusion weighted imaging with apparent diffusion coefficient (ADC) is commonly used to diagnose NPC recurrence, our founding of no significantly different mean ADC values between residual and non-residual groups was consistent with previous reports where no clear and definitive pre- and post-treatment ADC cutoff values have been established for clinical practice [6, 14]. The mean ADC value varies between poorly-differentiated and moderately or well-differentiated tumors, attributed to the lower ADC values of malignant tumors, indicating of increased cell density [49]. However, it is essential to consider that pre-treatment mean ADC values may be influenced by tumor extracellular and intracellular proteins, potentially leading to overestimation [38]. Mean ADC values have shown limited predictive capabilities for the long-term outcome of nasopharyngeal carcinoma after radiotherapy and chemotherapy [6]. The mean ADC values, obtained from multiple b-value DWIs using a bi-exponential model may have the potential to predict the response after NPC treatment [7, 32]. However, DWI is substantially greatly affected influenced by the field artifacts, particularly at the air-bone interface around the nasopharynx, leading to reduced accuracy of ADC values. In contrast, our studies have demonstrated that magnetization transfer ratio (MTR) values outperformed ADC values in predicting the response to nasopharyngeal carcinoma after CRT treatment.

The combination model using MTR with APTw-SI and ADC values did not significantly improve predictive efficacy (APTw-SI + ADC model, MTR + APTw-SI model, MTR + ADC model, and MTR + APTw-SI + ADC model; all P > 0.05). Therefore, pre-treatment MTR values remain a convenient and more effective predictor of the short-term treatment outcomes for NPC patients after CRT in clinical settings.

Older age, advanced TNM stage, and invasion of adjacent structures are well-known features for patients with worse prognosis [50]. The common clinicopathological characteristics did not have significant effect on predicting the short-term treatment outcome of NPC patients after CRT in our study. This could be attributed to the relatively short follow-up time (4 to 10 months) and the focus on early treatment response rather than long-term relapse-free survival. Additionally, the majority of patients included presented with advanced AJCC III/IV stage progressive NPC, potentially impacting the predicted treatment outcome.

Despite the valuable insights provided by our study, several limitations must be acknowledged. The relatively small sample size of forty patients with nasopharyngeal carcinoma, though strictly controlled for confounding factors, may introduce selection bias. Additionally, the relatively short average follow-up time limits the evaluation of long-term outcomes after chemoradiotherapy. Future research will aim to analyze the feasibility of APTw-SI and MTR values using 3D CEST technology in predicting long-term outcomes. Finally, the variation in dosages and time points of chemotherapy and radiotherapy among patients could impact the results, though the basic drugs for induction chemotherapy were consistent (GP regimens: gemcitabine + cisplatin) to ensure treatment uniformity.

## Conclusions

The pre-treatment MTR value had better prediction performance than ADC values and the APTw-SI acquired by a 3D CEST MR imaging, and it is more likely to predict short-term treatment outcomes of NPC patients after chemoradiotherapy. This finding holds promise for clinical applications, but further research and larger studies are needed to validate and establish the significance of these findings in the prediction of long-term treatment responses and patient outcomes.

### Electronic supplementary material

Below is the link to the electronic supplementary material.

Supplementary Material 1

## Data Availability

The datasets generated or analyzed during the study are available from the corresponding author on reasonable request.

## Abbreviations

APT: Amide proton transfer
MT: Magnetization transfer
MTR: Magnetization transfer ratio
CEST: Chemical exchange saturation transfer
APTw-SI: Amide proton transfer weighted-signal intensity
NPC: Nasopharyngeal carcinoma
CRT: Chemoradiotherapy
DWI: Diffusion-weighted imaging
ADC: Apparent diffusion coefficient
ROC: Receiver operating characteristic curve
ROI: Region of interest
CR: Complete response
