# Supplementary material for: Amide proton transfer (APT) and magnetization transfer (MT) in predicting short-term therapeutic outcome in nasopharyngeal carcinoma after chemoradiotherapy: a feasibility study of three-dimensional chemical exchange saturation transfer (CEST) MRI
Source: Cancer Imaging. 2023 Sep 1;23:80. doi: 10.1186/s40644-023-00602-6 (PMC10474660; doi:10.1186/s40644-023-00602-6)
Supplement: Supplementary file 1 — Supplementary Material 1 [file 40644_2023_602_MOESM1_ESM.docx]

| **Supplementary Table 1. Summary of magnetic resonance imaging parameters.** | | | | | | |
| --- | --- | --- | --- | --- | --- | --- |
| **Sequence** | **Ax T1WI IDEAL** | **Ax T2WI IDEAL** | **Ax rFOV DWI** | **IDEAL IQ, B0 measurement** | **Cube CEST, Phase Cycle RF** | **Ax T1WI IDEAL +C** |
| **Pulse Sequence** | 2D FSE-XL | 2D FSE-XL | 2D Spin Echo | 3D IDEAL IQ | 3D CUBE | 2D FSE-XL |
| **Repetition time (ms)** | 724.0 | 5317.0 | 2586.0 | 6.3 | 3000.0 | 724.0 |
| **Echo time (ms)** | 12.3 | 68.0 | 68.9 | 2.6 | 15.0 | 9.2 |
| **Flip angle (degree)** | 111 | 111 | N/A | 3 | N/A | 111 |
| **Number of Echoes** | 1 | 1 | 1 | 6 | 1 | 1 |
| **Echo Train Length** | 4 | 16 | N/A | 2 | 70 | 4 |
| **Field of view(cm)** | 24.0×24.0 | 24.0×24.0 | 18.0×9.0 | 20.0×15.0 | 20.0×15.0 | 24.0×24.0 |
| **Slice thickness (mm)** | 4.0 | 4.0 | 4.0 | 8.0 | 8.0 | 4.0 |
| **Slice Spacing (mm)** | 1.0 | 1.0 | 0 | 0 | 0 | 1.0 |
| **Number of slices** | 36 | 36 | 10 | 10 | 10 | 36 |
| **b factors (s/mm^2^)** | N/A | N/A | 0, 50, 800 | N/A | N/A | N/A |
| **Matrix** | 320×256 | 320×256 | 128×96 | 128×96 | 128×96 | 320×256 |
| **NEX** | 2 | 1 | 1, 10 | 1 | 1 | 2 |
| **Total scan time (min:sec)** | 1 min 53sec | 1 min 52sec | 1 min 17sec | 21sec | 4 min 56sec | 3 min 55sec |
| **Note:** CEST: Chemical exchange saturation transfer, IDEAL-IQ: Iterative Decomposition of water and fat with Echo Asymmetry and Least squares estimation, CUBE: three-dimensional fast spin-echo with an extended echo train acquisition. | | | | | | |
